# Formulation and Characterization of Ethyl Cellulose-Based Patches Containing Curcumin-Chitosan Nanoparticles for the Possible Management of Inflammation via Skin Delivery

**DOI:** 10.3390/gels9030201

**Published:** 2023-03-06

**Authors:** Asif Nawaz, Muhammad Shahid Latif, Muhammad Khurshid Alam Shah, Tarek M. Elsayed, Saeed Ahmad, Hamid Ali Khan

**Affiliations:** 1 Advanced Drug Delivery Lab, Gomal Centre of Pharmaceutical Sciences, Faculty of Pharmacy, Gomal University, Dera Ismail Khan 29050, Pakistan; 2 Pharmaceutical Technology Department, Faculty of Pharmacy, Sultan Zainal Abidin University, Besut Kampus, Besut 22200, Malaysia; 3 Institute of Biotechnology and Microbiology, Bacha Khan University, Charsadda 24420, Pakistan; 4 Directorate of ORIC, Bacha Khan University, Charsadda 24420, Pakistan

**Keywords:** chitosan, curcumin, nanoparticles, patches, inflammation and skin delivery

## Abstract

Curcumin, a natural phenolic compound, exhibits poor absorption and extensive first pass metabolism after oral administration. In the present study, curcumin-chitosan nanoparticles (cur-cs-np) were prepared and incorporated into ethyl cellulose patches for the management of inflammation via skin delivery. Ionic gelation method was used for the preparation of nanoparticles. The prepared nanoparticles were evaluated for size, zetapotential, surface morphology, drug content, and % encapsulation efficiency. The nanoparticles were then incorporated into ethyl cellulose-based patches using solvent evaporation technique. ATR-FTIR was used to study/assess incompatibility between drug and excipients. The prepared patches were evaluated physiochemically. The in vitro release, ex vivo permeation, and skin drug retention studies were carried out using Franz diffusion cells and rat skin as permeable membrane. The prepared nanoparticles were spherical, with particle size in the range of 203–229 nm, zetapotential 25–36 mV, and PDI 0.27–0.29 Mw/Mn. The drug content and %EE were 53% and 59%. Nanoparticles incorporated patches are smooth, flexible, and homogenous. The in vitro release and ex vivo permeation of curcumin from nanoparticles were higher than the patches, whereas the skin retention of curcumin was significantly higher in case of patches. The developed patches deliver cur-cs-np into the skin, where nanoparticles interact with skin negative charges and hence result in higher and prolonged retention in the skin. The higher concentration of drug in the skin helps in better management of inflammation. This was shown by anti-inflammatory activity. The inflammation (volume of paw) was significantly reduced when using patches as compared to nanoparticles. It was concluded that the incorporation of cur-cs-np into ethyl cellulose-based patches results in controlled release and hence enhanced anti-inflammatory activity.

## 1. Introduction

Curcumin is a hydrophobic polyphenol chemically known as diferuloyl-methane, derived from the herb *Curcuma longa* L. (family: Zingiberaceae) [1]. Curcumin has a wide range of beneficial effects and used traditionally for treatment of several diseases due to its wide spectrum of biological and pharmacological activities [2]. Curcumin has well reported multifunctional properties, including anti-inflammatory, anti-oxidant, anti-microbial, hypocholesterolemic, and anti-carcinogenic activities [3]. Anti-inflammatory activity of curcumin is due to regulating inflammatory signaling pathways and inhibiting the production of inflammatory mediators and thereby treating inflammatory diseases [4]. Curcumin can also regulate the Janus kinase/Signal transducer and activator of transcription inflammatory signaling pathway for the management of inflammation [5].

Major drawbacks associated with curcumin therapeutic applications include its poor aqueous solubility, poor absorption, rapid metabolism, and low stability [6]. The lipophilic molecules are more advantageous as compared to hydrophilic molecules and these drawbacks can be removed by converting curcumin into patch. Therefore, topical application of curcumin on the inflamed site can offer the advantage of delivering a drug directly to the disease site and producing its local effect. The poor aqueous solubility of curcumin also limits its skin permeation/topical application [7].

Topical drug delivery is gaining great attention in the field of pharmaceutical industry [8]. Skin is the largest organ of human body and stratum corneum, and the outer most layer of skin acts as main barrier against the entry of substances into the body [9].

Different techniques were implied to enhance the skin permeability of drugs/active pharmaceutical ingredients. One of the approaches is to encapsulate the drug into nanoparticles, which increases the drug permeation through the skin [10]. Chitosan is widely used polymer for nanoparticles. Chitosan is a naturally derived polymer having biocompatibility, biodegradability, mucoadhesivity, and permeation enhancement activity [11].

Furthermore, nanoparticles made from chitosan shows permeation enhancement, which favors their use as carrier in transdermal/topical delivery [12]. Chitosan-curcumin nanoparticles were also prepared by Duse et al., 2018 for photodynamic therapy, and found that incorporation of curcumin into chitosan nanoparticles helps in destruction of tumor cells more efficiently than curcumin alone [13]. Curcumin was also incorporated into chitosan nanoparticles for improved wound healing activity [14,15].

Previously, curcumin was topically used for the management of sprain and osteoarthritis [16]. In the present study curcumin chitosan nanoparticles were prepared for improved anti-inflammatory activity. The prepared nanoparticles were then incorporated into patches in order to avoid premature release of nanoparticles/curcumin at the surface of skin. Transdermal patches were used to delivery drug topically at a controlled rate. Patches were prepared using polymers either natural or synthetic. The patch is considered as the backbone of topical delivery systems [17]. Polymers used in the formulation of patches have desirable properties, e.g., hydrophilicity, biocompatibility, swelling ability, and controlled drug release rate [18].

In the current study, curcumin-chitosan nanoparticles were prepared and incorporated into ethyl cellulose based transdermal patches for the controlled delivery of curcumin into the skin for the possible management of the inflammation.

## 2. Results and Discussion

### 2.1. FTIR Analysis

**Figure 1:**
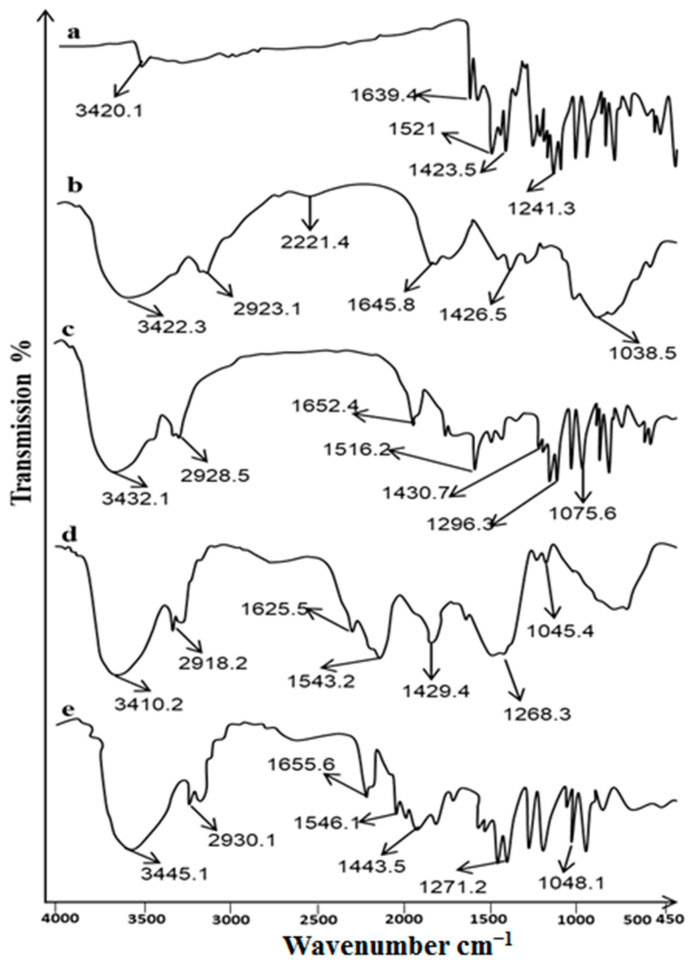
ATR−FTIR spectra of (**a**) Curcumin, (**b**) Chitosan, (**c**) Cur─Cs─Np, (**d**) Ethyl cellulose and (**e**) Cur─Cs─Np─P.

The ATR-FTIR spectra of drug (Curcumin), excipients/polymers (chitosan, ethylcellulose), and formulation (nanoparticles and patch) are shown in Figure 1. The peaks observed at 3420.1 cm^−1^ (the phenolic O-H stretching vibration), 2926 cm^−1^ (CH_2_ stretching vibration), 1639 cm^−1^ (assigned to the response of C=O (ketone) stretching vibration), and 1521 cm^−1^ (C=O and C=C characteristic vibrations) represents the characteristic peaks of curcumin [19]. The other peaks were observed at 1423 cm^−1^ representing enolic COH bending vibration, and 1241 cm^−1^ represents C-O stretching vibrations. The chitosan characteristic peaks were observed at 3422, 2923, 1645, and 1426 cm^−1^. The ATR-FTIR spectrum of Cur-Cs-Np shows the characteristic peaks of drug (curcumin) and polymer (chitosan) with minor shifting and decrease intensity, indicating absence of interaction. The slight variation in the peaks is due to the presence of some bonds (H-bonds, Vander wall forces) that result in entrapment of curcumin within nanoparticles. The patch ATR-FTIR spectra showed no change in the characteristic peaks of curcumin. This is due to entrapment/loading of curcumin into the polymer matrix of the patch.

### 2.2. Preparation and Characterization of Nanoparticles

**Figure 2:**
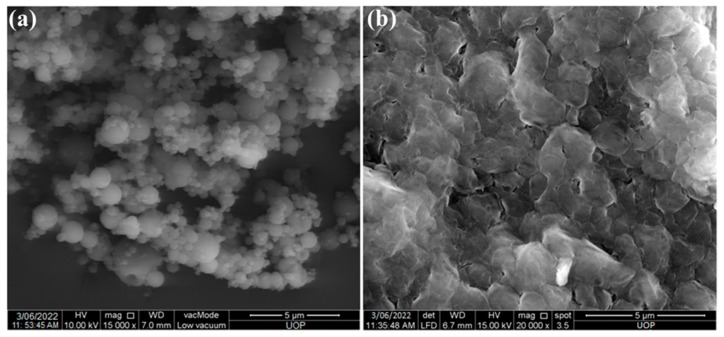
SEM image of (**a**) nanoparticles (Cur-Cs-Np) and (**b**) Patch (Cur-Cs-Np-P).

Curcumin formulation into nanoparticles improves its efficacy and stability. Chitosan was used as carrier for the formulation of nanoparticles. The average size of prepared nanoparticles was in the range of 203–229 nm (Table 1). The size of blank nanoparticles was smaller than the drug-loaded nanoparticles. The size of nanoparticles is important parameter as it affects the release profile, stability and biological performance of nanoparticles [12]. The zeta potential of prepared nanoparticles ranges between 25 to 36 mV. The positive zetapotential is attributed the presence of cationic polymer chitosan. The zeta potential value of curcumin loaded nanoparticles was slightly lower than the blank nanoparticles, this is due to curcumin occupies the +ve charge of chitosan resulting in lower surface charge of nanoparticles. The zeta potential value greater than +30 mV provides good stability and ability to attach/interact with negatively charged biological membranes. The prepared nanoparticles are spherical and having smooth surface as shown in Figure 2a. The entrapment efficiency of prepared nanoparticles was 59.3% and drug content was 53.2 ± 3.32% (Table 1). The hydrophobic nature and larger size of curcumin resulted in low % EE.

### 2.3. Preparation and Characterization of Patches

The patches were prepared using solvent evaporation technique. Blank, curcumin and Cur-Cs-Np loaded patches were prepared. The physicochemical characterization of prepared patches is shown in Table 2. The prepared patches were inspected visually for its physical appearance, and found to be smooth, homogeneous, and flexible. The surface morphology of the Cur-Cs-Np loaded patches are shown in Figure 2b. The prepared patches have some pores on the surface which helps in initial release of the nanoparticles. The pH of the formulated patches was in the range of 5.9–6.3. This pH range is in the acceptable range as the skin pH ranges from 5.5 to 6.5 [21]. The thickness of the patch ranges in between 0.73 to 0.84 mm. The thickness of the patches slightly increases with the incorporation of Cur-Cs-Np. Similarly, the weight of the patches also increased with the addition of Cur-Cs-Np. All the prepared patches showed folding endurance greater than 60, which suggests that patches will not easily break or damaged when applied on skin. The tensile strength value of prepared patches ranges in between 11.23 to 12.65 kg/cm^2^, indicating sufficient tensile strength with no significant difference. The % moisture content of all prepared patches ranges from 8.38 to 10.32%. The moisture content slightly increases with the addition of Cur-Cs-Np in the patches, which was due to the presence chitosan along with ethyl cellulose.

### 2.4. In Vitro Release

**Figure 3:**
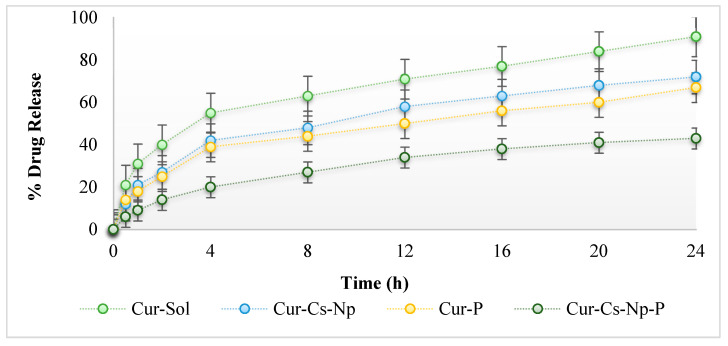
In-vitro release profile of curcumin. Note: Cur-Sol (curcumin solution), Cur-Cs-Np (curcumin chitosan nanoparticles), Cur-P (Curcumin patch), Cur-Cs-Np-P (curcumin chitosan nanoparticle patch).

Figure 3 shows the release profile of curcumin from nanoparticles and patch formulation. Phosphate buffer pH 5.5 was used to measure the curcumin release. Release of curcumin from nanoparticles was initially fast during the first 4 h, followed by sustained release, revealing a biphasic process. An initial burst release of curcumin which is adsorbed on the surface of the nanoparticles and further sustained release of entrapped curcumin was observed. Burst release of curcumin helps in topical/local disease management. Chitosan is a natural rate controlling polymer and helps in sustained release of drug. The drug encapsulated inside the chitosan matrix releases slowly over 24 h. The controlled/slower release of curcumin is due to entrapment of curcumin inside nanoparticles and longer diffusion path to follow [20]. Approximately 70% of curcumin was released from nanoparticles within 24 h. The release of curcumin from nanoparticles was significantly higher than the patch (ANOVA; *p* < 0.05). The nanoparticles incorporation into patches results in sustained release of curcumin as well as the release duration is extended to 36 h. The curcumin nanoparticles were faster released from prepared patches, followed by sustained release. The polymers used in the formulations of patches reduced the release of nanoparticles, which ultimately reduces the release of curcumin. Patch reduces the release of drug, as curcumin has to diffuse through the chitosan and patch polymers. This release profile was important to achieve the high concentration gradient required for successful topical delivery.

### 2.5. Ex Vivo Permeation and Skin Drug Retention

**Figure 4:**
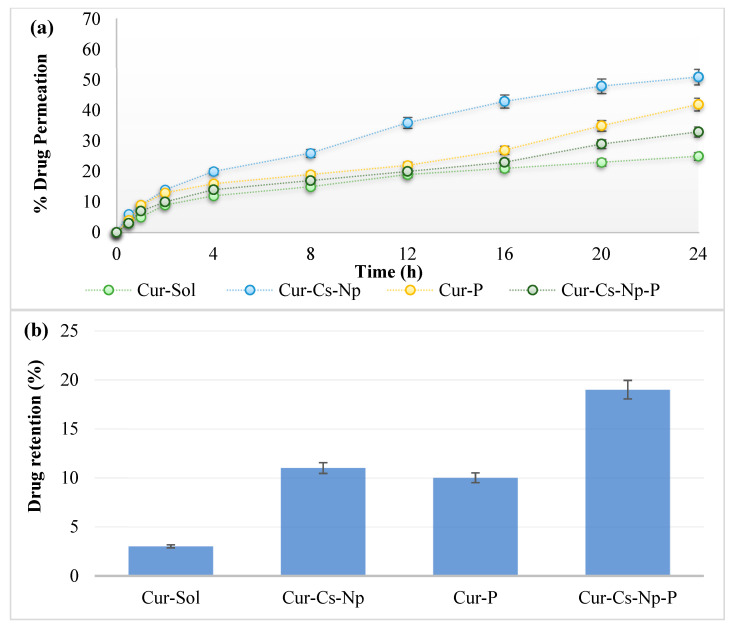
(**a**) Ex vivo permeation and (**b**) Skin drug retention of curcumin.

The ex vivo permeation and skin drug retention studies of nanoparticles and patches were performed using rats’ skin and Franz diffusion cell. Figure 4 shows the permeation profile of nanoparticles and patches. The permeation of cur-cs-np was slightly higher (ANOVA; *p* < 0.05) than the Cur-P and Cur-Cs-Np-P. Chitosan acts as permeation enhancer by reversibly altering the skin proteins and lipids, resulting in higher permeation of drug [22]. Skin permeability depends on nanoparticle size and chitosan content [23]. The drug release was higher with nanoparticles compared to patches and permeation follows the same trend, whereas skin drug retention was found to be maximum for the patches (ANOVA; *p* < 0.05). The drug release from nanoparticles was reduced by incorporating the nanoparticles into patch, which results in the permeation of nanoparticles without releasing the drug to the skin. The presence of nanoparticles inside the skin results in interaction with negatively charged lipids, which supports higher skin drug retention. Higher skin drug retention is helpful for the management of local disease and inflammation.

### 2.6. Anti-Inflammatory Studies

**Figure 5:**
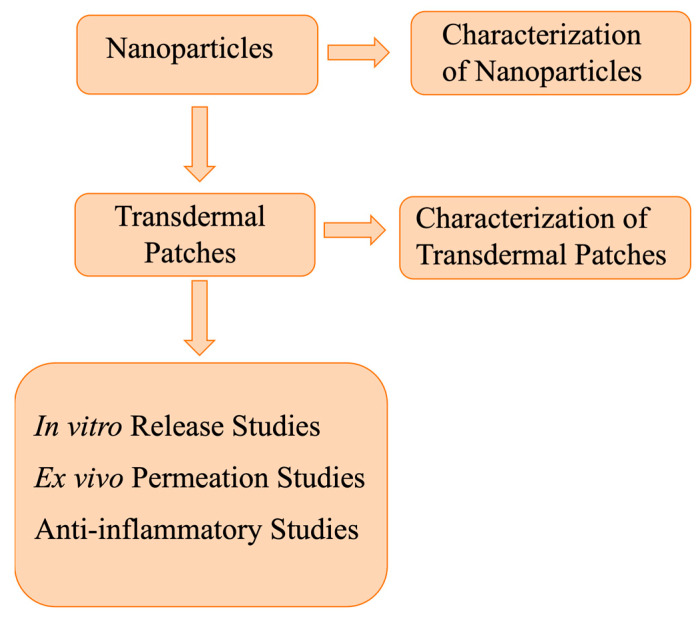
Scheme of the experimental procedure.

Anti-inflammatory results of prepared nanoparticles and patches were shown in Figure 5. Male Sprague Dawley rats were used in this study as inflammation is 3–5 times more common in female as compared to males. Curcumin is well documented as potential anti-inflammatory agent. Anti-inflammatory activity of curcumin was enhanced by incorporation into chitosan nanoparticles. It was evident from the results that maximum percent inhibition in paw volume was observed with nanoparticles incorporated patches (ANOVA; *p* < 0.05). Patches prominently inhibit the inflammation/edema as compared to nanoparticles and control group. Cur-Cs-Np also reduces the edema volume as compared to control but lower than the patches. This was due to higher drug deposition in the skin layers in the case of patches as compared to nanoparticles. The higher concentration of drug in the deeper layers of the skin helps to overcome skin infection and inflammation. The duration of reduction in the paw edema volume was higher in the case of patches as compared to nanoparticles. This was due to the continuous and prolonged release of curcumin from the patches, which results in the continuous supply of curcumin to the inflamed area. The nanoparticles incorporated patches delivers the therapeutically required amount of drug at the target site more efficiently than the nanoparticles. Therefore, the topical delivery of curcumin using nanoparticles incorporated patches supports the management of inflammation.

## 3. Conclusions

Curcumin nanoparticles were prepared using ionic gelation method. The prepared nanoparticles were then incorporated into the patches. The use of curcumin-chitosan nanoparticles may take advantage of the functional properties of curcumin, chitosan nanoparticles in an additive way to fight against inflammation. The in-vitro release of curcumin was controlled by incorporating nanoparticles into transdermal patches. The skin drug retention of nanoparticles incorporated patches was found to be higher than nanoparticles and patch alone. Higher skin drug retention will support the better management of local disease such as inflammation. This was also confirmed by the anti-inflammatory results. Curcumin-chitosan nanoparticles incorporated into a patch could be a new powerful drug delivery for local skin diseases such as inflammation.

## 4. Materials and Methods

### 4.1. Material

Curcumin (98% Pure) was purchased from RMY Exporter, Mumbai, India. Chitosan having molecular weight (MW: 350,000 Da, deacetylation degree > 75%), Triphenyl phosphate (TPP), and poly(vinyl pyrrolidone) (PVP) were supplied by Sigma-Aldrich, St. Louis, MO, USA. Ethyl cellulose was purchased from Dow Chemical Company, Washington. λ-Carrageenan (Sigma-Aldrich, St. Louis, MO, USA). All the chemicals used were of analytical grade.

### 4.2. ATR-FTIR Analysis

ATR-FTIR spectra of curcumin, chitosan, ethyl cellulose, nanoparticles, and patches were examined using the ATR-FTIR spectrometer (PerkinElmer, 940 Winter St Waltham, MA, United States). The samples were placed on the sample holder without any processing and scanned between 4000 and 400 cm^−1^. Spectra were recorded in triplicates and results were averaged [17].

### 4.3. Synthesis of Curcumin-Loaded Chitosan Nanoparticles

Chitosan-curcumin nanoparticles were prepared using the ionic gelation method, as previously described by Basit et al., 2020 with slight modifications [15]. TPP was used as crosslinker and its ratio with chitosan was 1:3. Curcumin was first dissolved in ethanolic solution (water: ethanol; 1:1) at a concentration of 1 mg/mL (1% *w/w*). Chitosan (0.5% *w/w*) solution was prepared in 2% *v/v* aqueous acetic acid, and its pH was adjusted to 5 with 2 M NaOH. Then, the curcumin solution was added in to the chitosan solution and followed by the dropwise addition of aqueous TPP solution (0.125% *w/v*) under constant magnetic stirring (1000 rpm). The nanoparticles were spontaneously formed and harvested by centrifugation at 10,000 rpm for 1 h. The pellet formed was then re-dispersed in deionized water and freeze dried (Freezone loor top freeze dryer, Labconco, Kansas City, MO, USA) at −50 °C for 24 h.

#### 4.3.1. Size and Zeta Potential Analysis

Average particle size distribution and zeta potential of the prepared nanoparticles was determined by dynamic light scattering (DLS) using a Malvern Zetasizer instrument (Malvern Instruments, Malvern, Worcestershire, UK). The prepared NPs were taken and dispersed in distilled water to a final concentration of 1% and ultrasonicated before the measurement. Three measurements were conducted for each sample and results were averaged [24].

#### 4.3.2. Surface Morphology

The surface morphology of the prepared nanoparticles and nanoparticles incorporated patch was determined using SEM (Zeiss EVO40; Carl Zeiss, Cambridge, UK). Briefly, nanoparticles were taken and spread on a double-sided conductive tape and the surface was coated with gold under high vacuum. The images were taken at different resolutions [25].

### 4.4. Preparation of Nanoparticles Loaded Patches

Nanoparticles loaded patches were prepared using solvent casting technique. Ethyl cellulose was used as patch forming polymer. Polymer and excipients were weighed precisely using analytical balance (Shimadzu AX 200, Kyoto, Japan) and dissolved in the solvent system (comprised of equal amount of 10 mL of ethanol and distilled water) using magnetic stirrer. The prepared nanoparticles were suspended in distilled water. The NPs aqueous phase was added to the polymer solution under continuous stirring. PEG-400 was added as plasticizer. The solution was sonicated using sonicator (D-78224, Singen, Germany) and carefully poured into petri-dishes. The Petri dishes were dried in oven in dark and at 37 °C temperature. The final prepared patches were kept in desiccator until further use [26].

### 4.5. Characterization of Patches

#### 4.5.1. Physicochemical Characterization of Patches

The prepared patches were evaluated physiochemically as described by our previously reported methods [27]. Briefly, prepared patches were physically inspected for color, smoothness, and clarity. The thickness of the patches was measured using vernier calipers (Germany) at six different places and results were averaged. The weighed uniformity of all patches was determined using an analytical weighing balance (Shimadzu AX 200, Kyoto, Japan). The surface pH of the formulated patches was measured by placing a pH meter (InoLab^®^, Xylem Analytics, Weilheim 82362, Germany) rod on the surface of the patch and reading was recorded. Folding endurance was determined by folding the patch several times at the same point till it breaks. All the experiments were conducted in triplicates and results were averaged.

#### 4.5.2. Drug Content

Drug content of the prepared patches was evaluated using UV visible spectrophotometer (Shimadzu 1601, Kyoto, Japan). A patch having area of 1 cm^2^ was placed in a volumetric flask filled with phosphate buffer (pH 7.4) and sonicate for 8 h. The solution was then centrifuged, and supernatant was analyzed on UV spectroscopy. The pellet was dissolved in 1% *v/v* acetic acid solution and analyzed using a UV visible spectrophotometer at 425 nm wavelength. Both the readings were averaged and drug content was calculated using calibration curve [28].

#### 4.5.3. Tensile Strength and Percent Elongation

Pulley system was used for the evaluation of mechanical properties of prepared patches. The initial length of patch was measured using scale. One side of the prepared patch was tied to the hook and another side of the patch was tied to the rope. The rope was crossed over a pulley and attached to the weighing pan. Weight was gradually added to the weighing pan until creak or break appears in prepared patch. The total weight of weighing pan was calculated for the value of tensile strength. The value for percent elongation was evaluated using thread pointer method [29].

Tensile strength value was determined using following equation.

Tensile Strength = F/(a·b(1 + L/I))(1)

where F indicates total amount of force required to break patch, a indicates the width of patch (cm), and b indicates thickness of patch (cm). L indicates length of patch (cm), and I indicate elongation of patch before patch breakage (cm). The following equation was used for the evaluation of patch percent elongation [30]:

% Elongation = (Lf − Li)/Li × 100(2)

where Li indicates initial length of patch and Li indicates final length of patch.

### 4.6. In Vitro Drug Release Study

The in vitro release of nanoparticles and patches was performed using Franz Diffusion Cell Apparatus (Perme-Gear, Hellertown, PA, USA) and synthetic membrane (Tuffryn membrane; diameter 2.5 mm and pore size 0.45 µm). The membrane was fixed between donor and receptor compartment. The receptor compartment was filled with phosphate buffer (pH 5.5) and 1.5% polysorbate 80 in-simulation to skin pH. The temperature of the receptor compartment was maintained at 32 ± 0.5 °C and stirred at 100 rpm. The donor compartment was charged with Cur-Sol (curcumin solution), Cur-Cs-Np (curcumin chitosan nanoparticles), Cur-P (Curcumin patch), and Cur-Cs-Np-P (curcumin chitosan nanoparticle patch), each containing 5 mg of drug. Then, 2 mL sample was collected at specified time intervals, i.e., 0.5, 1, 1.5, 2, 4, 8, 12, 16, and 24 h, respectively, and replaced with equal volume of fresh buffer (pH 5.5) to maintain the sink condition. The collected samples were analyzed using UV visible spectrophotometer at 425 nm wave length [31].

### 4.7. Ex Vivo Drug Permeation and Retention Study

Ex-vivo studies of nanoparticles and patches were carried out across rat’s skin. The procedure performed to extract skin from rats was approved from Ethical Review Board, Gomal Centre of Pharmaceutical Science, Faculty of Pharmacy, Gomal University, Dera Ismail Khan, KP, Pakistan. Male Sprague Dawley rats weighing 200–250 g were sacrificed using a cervical dislocation method. Hairs were removed from the dorsal region of the rats using electric trimmer. The skin was then removed surgically, washed with 0.9% sodium chloride solution, and was kept at −20 ± 1 °C. The rat’s skin was then mounted between donor and receptor compartment of Franz diffusion cell. The receptor compartment was filled with phosphate buffer (pH 7.4) and temperature was maintained at of 37 ± 2 °C. The receiver medium was stirred at 100 rpm. The donor compartment was loaded with nanoparticles/patch containing 5 mg of drug. Hence, 2 mL aliquots were taken at specified time intervals, i.e., 0.5, 1, 1.5, 2, 4, 8, 12, 16, and 24 h, respectively, and replaced with fresh buffer in order to maintain sink condition. The samples were analyzed using a UV visible spectrophotometer at 425 nm wave length [32].

Ex-vivo permeation study was followed by skin drug retention analysis. The skin obtained from ex vivo study was dried with tissue paper, cut into small pieces, and suspended in phosphate buffer pH 7.4. Then, 5 mL of methanol was added for extraction of drug. The skin was the homogenized using tissue homogenizer and then centrifuged. The supernatant was taken and analyzed using a UV visible spectrophotometer at 425 nm wavelength, and the results obtained were calculated and averaged (mean ± SD).

### 4.8. Anti-Inflammatory Activity

Anti-inflammatory activity of prepared nanoparticles and patches were carried out using male Sprague Dawley rats, weighing 200–250 g and protocol was approved from ERB, GCPS, Gomal University Pakistan. The rats were divided into three groups (Group A-Control, Group B-Nanoparticles and Group C-NPs loaded Patch). All the rats were given access to food and water, and temperature was maintained at 25 ± 2 °C with humidity 55%. The natural light and dark cycle were also employed. Inflammation was developed by carrageenan injection in the right hind paw, whereas normal saline (non-pyrogenic) was injected in the left hind paw to act as control. The prepared nanoparticles and patch were applied on the inflamed paw. Paw thickness was measured at 0 h, and at 1, 2, 4, 8, 16, and 24 h using a digital plethysmograph. Control thickness was compared with the inflamed paw for each group [33].

### 4.9. Statistical Analysis

Statistical analysis was performed using SPSS version 18. ANOVA was used for analysis.

## Figures and Tables

**Table 1:** Characterization of nanoparticles.

F. Code	Size [20]	Zeta Potential (mV)	PDI	Drug Content (%)	%EE
Cs-Np	203.1 ± 6.95	+36.3 ± 1.25	0.27 ± 0.12	----	-----
Cur-Cs-Np	229.4 ± 9.53	+25.8 ± 1.56	0.29 ± 0.11	53.2 ± 3.32	59.3 ± 2.98

Data were expressed as mean ± SD, *n* = 3.

**Table 2:** Physicochemical characterization of prepared patches.

F. Codes	pH	Thickness (mm)	Weight Variation (mg)	Folding Endurance	Tensile Strength kg/cm^2^	% Moisture Content	% Drug Content
Blank-P	5.9	0.73 ± 0.14	85.67 ± 0.12	67 ± 2.35	11.23 ± 0.56	8.38 ± 0.71	-----
Cur-P	6.2	0.78 ± 0.21	88.54 ± 0.25	60 ± 2.67	11.13 ± 0.72	9.76 ± 0.86	83.51 ± 2.92
Cur-Cs-Np-P	6.3	0.84 ± 0.27	91.98 ± 0.29	58 ± 2.14	12.65 ± 0.86	10.32 ± 0.95	85.73 ± 2.49

Data were expressed as mean ± SD, *n* = 3.

## Data Availability

Not applicable.
